# Health workers’ values and preferences regarding contraceptive methods globally: A systematic review^☆^

**DOI:** 10.1016/j.contraception.2022.04.012

**Published:** 2022-07

**Authors:** Komal S. Soin, Ping Teresa Yeh, Mary E. Gaffield, Christina Ge, Caitlin E. Kennedy

**Affiliations:** a Department of International Health, Johns Hopkins Bloomberg School of Public Health, Baltimore, MD, United States; b Department of Family Medicine and Community Health, University of Hawaii, John A. Burns School of Medicine, Aiea, HI, United States; c Contraception and Fertility Care Unit, Department of Sexual and Reproductive Health and Research, World Health Organization, Geneva, Switzerland; d Department of Obstetrics and Gynecology, Anne Arundel Medical Center, Annapolis, MD, United States

**Keywords:** Contraception, Health Care providers, Health workers, Preferences, Systematic review

## Abstract

**Objective:**

We sought to systematically review the literature on health workers’ values and preferences related to contraceptive methods.

**Study design:**

As part of a larger review, we searched ten electronic databases for published articles from January 1, 2005 through July 27, 2020. We included studies that reported qualitative or quantitative data from the perspective of health workers providing family planning services globally.

**Results:**

Forty-one studies met our inclusion criteria. These studies included 12,643 health workers and were conducted in 27 countries. Health worker values and preferences for contraceptive methods were affected by factors related to contraceptive method characteristics (e.g., bleeding pattern and convenience), the contraceptive user (e.g., medical history, parity), and the health worker themselves (e.g., training, environment). Differences were also noted between various professions/specialties (e.g., comfort level with contraceptive methods, depth of experience). While contraceptive counseling and provision were influenced by health worker values and preferences, they were also affected by health worker misconceptions and biases.

**Conclusion:**

Health worker values and preferences for contraception are affected by the client's history, medical eligibility, and the health worker context. Provision of contraception that is affected by harmful bias towards certain populations or about certain methods can negatively affect patient-centered care. Future work should address knowledge gaps and health worker biases by improving and standardizing education and training globally, to ensure high-quality, rights-based, and patient-centered contraceptive services.

## 1 Introduction

Improving access to contraceptive methods and supporting informed choice in the use of these methods is key to the health and well-being of mothers and families [1,2]. Health workers are on the frontlines of providing family planning services to clients (e.g., counseling and contraception). They are responsible for equipping clients with thorough and accurate information about contraception to help them come to an informed decision. However, personal biases can impede the ability of health workers to fully assess client needs and create barriers to choice [3]. Understanding health worker counseling and prescribing practices along with their values and preferences regarding contraception is a fundamental component of overall quality of care, which has been conceptualized as including both the quality of the provision of care and the quality of care as experienced by clients [4].

Health workers who provide contraceptive counseling include physicians in multiple specialties (e.g., family medicine, pediatrics, obstetrics and gynecology (Ob/Gyn), emergency medicine, internal medicine), nurse practitioners, physician assistants, midwives, and community health workers, among other cadres [5]. They come from many educational backgrounds, leading to varying competence and comfort levels in contraception provision. In addition, studies have documented health worker biases regarding specific contraceptive methods and their use in certain populations: health worker contraception counseling practices can be affected by their perception of pregnancy risk, understanding of contraceptive eligibility for certain populations (including medical eligibility), workplace setting, and prior training [6], [7], [8]. Health worker racial and ethnic biases may also impact their interactions with clients [9,10]. A recent literature review found evidence of health worker bias based on client age, parity, and marital status, among other criteria; the most common health worker bias reported was bias against provision of various contraceptive methods to youth [3]. While many contraceptive users desire autonomy in selecting a contraceptive method, many also value input from their health worker and seek comprehensive information regarding their options. It is important to therefore understand how health workers feel about different contraceptive methods, and how these perspectives may shape their interactions with clients and, ultimately, their clients’ method choices. Understanding these perspectives, and working to reduce instances of health worker bias, is therefore critical to ensuring client-centered contraceptive services.

We therefore sought to systematically review the literature on values and preferences for contraception among health workers. This work was conducted as part of a larger project to inform WHO contraceptive guidelines, which include considering the acceptability of interventions to health workers and other stakeholders among a range of other important factors [11].

## 2 Methods

This review was part of a larger set of reviews on values and preferences related to contraception among diverse populations globally [2]. The review was conducted according to PRISMA (Preferred Reporting Items for Systematic Reviews and Meta-Analyses) guidelines [12]. Below, we briefly describe the methods used to conduct the review on health worker contraceptive values and preferences, but a detailed description of the methods for the larger review is available elsewhere [1].

We included quantitative and qualitative studies that reported on values and preferences specifically among health workers. We included health workers of any cadre, including health extension workers, family planning counselors, nurses, midwives, physicians (primary care/general practice, internal medicine, Ob/Gyn, etc.), and hospital administrators. We included studies related to any combination of the following methods: combined oral contraceptive pills (COCPs), progestogen-only pills, combined patch, combined vaginal ring, progesterone vaginal ring, combined injectable contraceptives, depot medroxyprogesterone acetate (DMPA), injectable norethisterone enanthate, levonorgestrel and etonogestrel implants, emergency contraceptive pills, copper-bearing intrauterine devices (Cu IUD), levonorgestrel-releasing intrauterine devices (LNG IUD), copper-IUD for emergency contraception, physical and chemical barrier methods, fertility awareness-based methods, lactational amenorrhea, coitus interruptus, and female and male sterilization. We excluded case studies, case series, review articles, editorials, and letters, focusing instead on primary research. To focus on the peer-reviewed literature, we excluded conference abstracts and posters, as well as theses and dissertations. Studies had to describe some aspect of values and preferences, defined by Guyatt et al. as “goals, expectations, predispositions and beliefs that individuals have for certain decisions and their potential outcomes” [13], related to contraception. We excluded studies that solely reported knowledge, contraceptive prevalence, user characteristics, demographic factors associated with use or discontinuation, or contraceptive effectiveness. For health workers, we thus considered studies discussing their own perspectives on different contraceptive methods, their perspectives in relation to clients for whom those methods were appropriate, and counseling around those methods when it was interpreted as reflective of health worker values and preferences. We included studies from any country or setting and in any language. For studies published in a language other than English, we identified proficient speakers to abstract data.

We searched ten electronic databases for articles published between January 1, 2005 and July 27, 2020: PubMed, PsycINFO, Sociological Abstracts, CINAHL, Scopus, LILACS, WHO Global Health Libraries, Ovid Global Health, Embase, and POPLINE. Specific search terms are reported elsewhere [1]. We also conducted secondary searching of references of relevant review articles and included articles.

Reviewers screened and extracted data from studies as part of the larger global systematic review of contraceptive values and preferences using methods described elsewhere [1], including information such as author(s), year of publication, funding source, study location, study design, study population, contraceptive method(s), key results, and study rigor (assessed using an 8-item tool developed by the Evidence Project for quantitative studies [14] and a modified version of the Critical Appraisal Skills Programme checklist for qualitative studies [15]). Data abstraction was conducted in duplicate by 2 independent reviewers, and differences were resolved through discussion and consensus. KS and PTY screened studies that reported health workers as study participants for inclusion in this specific review. Using descriptive content analysis [16], KS and PTY reviewed coding across all included studies to compare across heterogeneous studies and capture relevant content areas. Because of the wide variety of study populations, study designs, and findings, meta-analysis was not possible.

## 3 Results

### 3.1 Search results

**Fig. 1:**
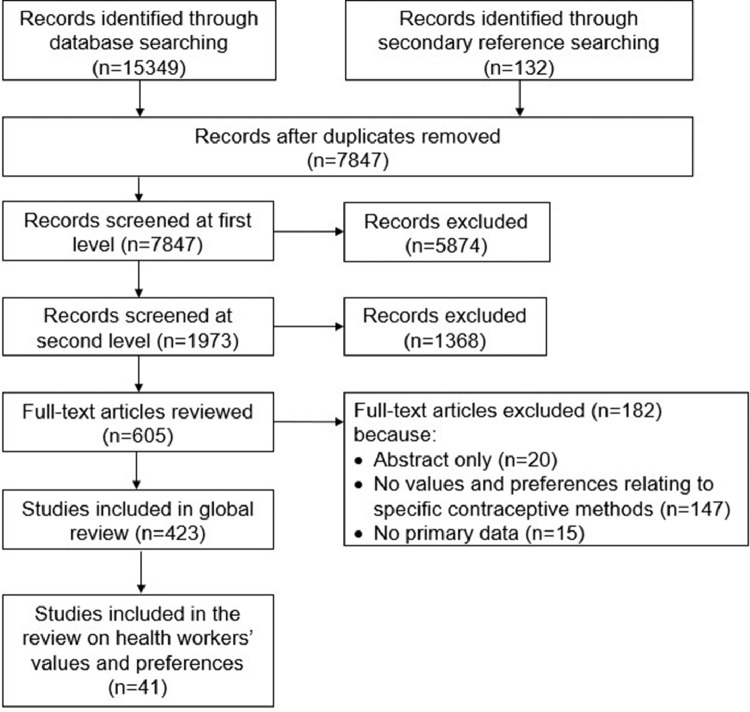
PRISMA flow diagram presenting the search and screening process for the global review on contraceptive method values and preferences from the perspective of health workers 2005-2020.

**Fig. 2:**
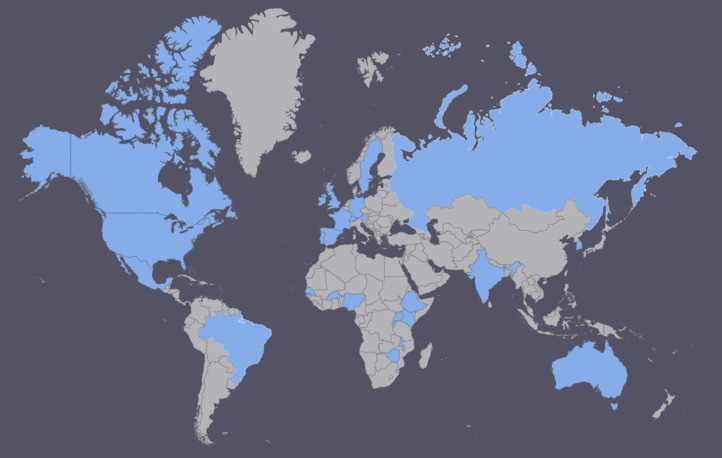
World map showing location of included studies presenting values and preferences from the perspective of health workers 2005-2020.

Through our systematic database search, we identified 15,349 potential articles; we found an additional 132 articles through other methods like secondary reference searching (Fig. 1). Of these, we included 41 articles reporting on values and preferences around contraceptive methods from the perspective of health workers in this review (Table 1) [17], [18], [19], [20], [21], [22], [23], [24], [25], [26], [27], [28], [29], [30], [31], [32], [33], [34], [35], [36], [37], [38], [39], [40], [41], [42], [43], [44], [45], [46], [47], [48], [49], [50], [51], [52], [53], [54], [55], [56], [57]. The 41 articles capture values and preferences of 12,643 health workers from 27 countries. Thirty-two percent of studies were conducted in low- and middle-income countries: with the remaining from high-income countries, mostly in Europe, Australia, and the United States of America (Fig. 2). Health workers included physicians from various specialties (e.g., Ob/Gyn, family medicine, general practitioner, internal medicine, and pediatrics), physician assistants, nurses including midwives and nurse practitioners, pharmacists, community health workers, social workers, health counselors and educators, health advocates, medical assistants, administrative directors, and office receptionists. Contraceptive methods described included COCPs, patch, injectable (e.g., DMPA), implant, hormonal IUD and Cu-IUD, male and female sterilization, male and female condoms, withdrawal, diaphragm, natural family planning (e.g., standard days method), vaginal gel, and emergency contraception. Of the included studies, 21 were quantitative, 16 qualitative, and 4 multi- or mixed-methods. Most quantitative studies were cross-sectional surveys, while qualitative studies predominantly used in-depth interviews and focus groups. Study rigor assessments are presented for qualitative studies in Appendix 1 and quantitative studies in Appendix 2.

**Table 1:** Summary characteristics of studies included in the global review on contraceptive method values and preferences from the perspective of health workers 2005-2020

Lead author year	Study location	Study design	Population and sample size	Contraceptive method(s)	Key results
Akers 2010	Pittsburgh, Pennsylvania, USA	Qualitative, Focus group discussions	*n* = 48 physicians, nurses, and pharmacists working in primary care clinics (8 FGDs)	pills, IUD, implant	Provider perceptions of challenges to counseling
Alem 2014	Adigrat Town, Tigray, Ethiopia	Qualitative, In-depth interviews	*n* = 6 family planning providers	long-acting vs short-acting methods	Family planning providers counseling practices and perceptions of patient preference for methods
Amouroux 2018	France	Quantitative, Cross-sectional	*n* = 605 new medical residents, general practitioners (GPs), and Ob/Gyn physicians	condom, thermal male contraception, hormonal male contraception, vasectomy, withdrawal	New providers knowledge and prescribing patterns of male contraception.
Benfield 2018	New York, USA	Quantitative, Serial cross-sectional	*n* = 115 post-partum and labor and delivery nurses	IUD, implant, DMPA	Nurses’ preference for LARC postpartum
Bitzer 2009	Basel, Bern, Zurich, Switzerland	Quantitative, Cross-sectional	*n* = 182 (156 gynecologists and 25 GPs)	combined oral contraceptive pills	Physicians' preference of different doses of COCs based on patients' medical conditions
Bombas 2012	Portugal	Quantitative, Cross-sectional	*n* = 303 gynecologists	combined hormonal contraceptives	Factors that influence contraception counseling, shared decision-making, and physician's perception of patient preferences regarding method choice, use, discontinuation.
Brunie 2019	Multi country: New Delhi, India; Ibadan, Nigeria	Qualitative, in-depth interviews	*n* = 20 family planning providers	patch	Acceptability of a new microarray patch in development
Buhling 2014	Germany	Quantitative, Cross-sectional	*n* = 1918 gynecologists	all hormonal methods	Physicians’ contraceptive recommendations for patients vs self/family
Burke 2014	Multi country: Mbour, Thies, Tivaouane, Senegal; Mubende and Nakasongola, Uganda	Qualitative, In-depth interviews embedded in a non-randomized controlled cohort study	*n* = 86 (67 community health workers, 20 clinic based health workers i.e. nurses and midwives)	Sub-Q vs IM Depo	Preference for Sub-Q vs IM DMPA formulation
Callahan 2019	Multi country: Burkina Faso; Uganda	Qualitative, In-depth interviews embedded in a multi-method study which included cross-sectional survey and focus group discussions with contraceptive users	*n* = 37 family planning providers	IUD-copper and hormonal, 2 types of implants, injectable, permanent sterilization	Providers thoughts on advantages vs disadvantages of various methods
Choi 2010	British Columbia, Canada	Quantitative, Cross-sectional	*n* = 235 family medicine physicians and residents, family medicine students, Ob/Gyn physicians and residents	Natural family planning	Physicians' perceptions and counseling practices on natural family planning methods.
Donnelly 2014	Maine, New Hampshire, Vermont, USA	Quantitative, Cross-sectional	*n* = 188 nurses, midwives, physicians' assistants (PA), medical doctors, other health workers such as counselors, social workers, clinic site manager	n/a	Comparison of patient's priorities in contraceptive decision-making vs health worker priorities for contraceptive counseling.
Garrett 2015	Australia	Qualitative, In-depth interviews	*n* = 15 GPs, nurses, medical directors of reproductive and sexual health organizations, sexual health educator, and health advocates	LARCs	Barriers to LARCs from health worker and client perspectives
Gemzell-Danielsson 2012	Multi-country: Australia, Brazil, Canada, France, Germany, South Korea, Mexico, Spain, Sweden, United Kingdom	Quantitative, Pre-post	*n* = 1001 Ob/Gyns, GPs, midwives	All methods	Health worker use of contraception and their recommendations
Kavanaugh 2013	USA	Qualitative, In-depth interviews and focus group discussions	*n* = 20. admin directors at publicly funded sites providing family planning services (IDIs); *n* = 37 physicians, educators, medical assistants, receptionists (6 FGDs)	LARCs: IUDs and implants	Advantages and disadvantages of LARCs from health worker and client perspectives
Keith 2014	Oromia Region, Ethiopia	Qualitative, Focus group discussions and in-depth interviews	*n* = 18 (8 physicians, 10 key informants); *n* = 46 nurses, pharmacists/drug shop owners, health extension workers (7 FGDs)	injectable	Health worker attitudes towards patient's self-injection of injectable contraceptive
Kelly 2016	Sydney, New South Wales, Australia	Qualitative, In-depth interviews	*n* = 15 physicians – contraceptive providers	LNG-IUS, pills, fertility awareness based methods, withdrawal	Physicians prescribing behavior of different methods of contraception.
Kilander 2017	Sweden	Qualitative, In-depth interviews	*n* = 21 (15 gynecologists, 6 midwives)	LARCs and other hormonal contraceptive methods (e.g. IUD/ LNG-IUS, pills), withdrawal, sterilization, implants	Contraception counseling in women seeking abortion.
Knox 2012	Australia	Quantitative, Cross-sectional	*n* = 162 GPs	Hormonal contraceptive methods, copper IUD	Physicians' thoughts on various forms of contraception based on benefits, side-effects, risks.
Larivaara 2010	St. Petersburg, Russia	Qualitative, In-depth interviews and observations	*n* = 16 Gynecologists (IDIs), *n* = 20 (observations of 46 clinic visits)	Vaginal ring, patch, pill, IUD, condom, withdrawal, rhythm method, vaginal douching	Physicians' views on methods of contraception and termination of pregnancy, their counseling methods, and prescribing behavior for different populations.
Lee 2019	South Korea	Quantitative, Cross-sectional	*n* = 946 Ob/Gyn, family medicine, internal medicine, pediatric physicians	EC	Physicians' thoughts on EC being prescription or behind the counter.
Madden 2010	Illinois, USA	Quantitative, Cross-sectional	*n* = 137 Ob/Gyn physicians	IUD	Physicians’ counseling and prescribing practices based on patient's parity, marital status, medical history.
Mantell 2011	New York City, NY, USA	Qualitative, In-depth interviews	*n* = 78 HIV/STI counselors and health educators, physicians, nurses, nurse practitioners (NP), peer/addiction/harm reduction counselors	female condom	Health worker knowledge and thoughts about female condom.
McLean 2017	San Francisco Bay Area, CA, USA	Mixed methods, Cross-sectional and in-depth interviews	*n* = 38 NP, certified nurse midwife, PAs, physicians	IUD, pills, condoms, cervical cap, diaphragm, injection, patch, sponge, sterilization	Health worker self-disclosure about personal contraception use during contraception counseling
Munsell 2009	Galveston, TX, USA	Quantitative, Cross-sectional	*n* = 126 PA students	EC	PA students’ thoughts on prescribing Plan B in future practice.
Newmann 2013	Migori, Rongo, Siba districts, Nyanza Province, Kenya	Mixed methods, Cross-sectional and in-depth interviews	*n* = 31 (18 clinical officers or nurses; 13 HIV counseling and testing counselors, community clinic health assistants, or health worker volunteers)	various methods including short and long acting methods	Health worker knowledge and views on family planning and pregnancy for HIV positive men and women.
Nguyen 2017	USA	Quantitative, Cross-sectional	*n* = 178 family planning providers - family medicine and Ob/Gyn	vasectomy, female sterilization	Physicians' views on vasectomy and counseling practices.
Oppelt 2017	Germany	Quantitative, Cross-sectional	*n* = 1089 gynecologists	LARCs	Effect of physician counseling on a client's contraceptive method choice.
Paul 2016	Central region, Uganda	Qualitative, In-depth interviews	*n* = 27 (10 physicians and 17 midwives)	general	Health worker contraceptive counseling practice based on their skill, education, and societal norms.
Philliber 2014	Colorado, Iowa, USA	Quantitative, Cross-sectional	*n* = 95 family planning providers: physicians, PAs, NPs, certified mid-wives	IUDs, implants	Health worker prescribing practices of LARCs based on how long they have been in practice.
Sulak 2006	USA	Quantitative, Cross-sectional	*n* = 551 primary care and Ob/Gyn physicians, NPs, PAs, midwives	Extended cycle OCPs	Health workers’ counseling and prescribing practices of extended oral contraceptive regimens.
Sweeney 2015	Galway, Republic of Ireland, UK	Qualitative, In-depth interviews	*n* = 27 (18 GPs, 9 pharmacists)	OCPs, LARCs	Physicians' and pharmacists' contraceptive counseling and prescribing practices for OCPs and LARCs.
Tolley 2014	Multi-country: Kenya, Rwanda	Qualitative, Focus group discussions and in-depth interviews	*n* = 46 health workers including nurses, counselors, and community health workers; policy makers (IDIs)	injectable	Health worker thoughts on what is important when creating a new injectable form of contraception.
Tyler 2012	USA: multiple sites	Quantitative, Cross-sectional	*n* = 1958. (635 office-based physicians (Ob/Gyn, family medicine, adolescent medicine) and 1323 Title X Clinic Health Workers (e.g., physicians, certified nurse midwife, NPs, PAs, nurses))	IUDs	Health worker misconceptions and prescribing practices with IUDs
Ujuju 2011	Rimi, Katsina, Kaita, Katsina state; Nkanu West, Enugu East, Igbo-Etiti, Enugu state, Nigeria	Qualitative, In-depth interviews	*n* = 12 (urses and family planning providers)	Standard days method	Health worker thoughts on standard days method.
Weisberg 2013	Australia	Quantitative, Cross-sectional	*n* = 162 GPs	implant, hormonal IUD	Physicians ranking of contraceptive characteristics (pluses and minuses).
Wellings 2007	United Kingdom	Quantitative, Cross-sectional	*n* = 321 GPs, NPs, nurses	LARCs, short acting, condom, diaphragm	Health worker thoughts about and prescribing practices of various methods of contraception.
Wiebe 2012	British Columbia, Canada	Mixed methods, Cross-sectional and in-depth interviews	*n* = 170 family medicine and Ob/Gyn practicing and resident physicians (155 surveys, 15 IDIs)	general	Physicians' thoughts on effect of contraceptive side effects on contraception discontinuation and counseling practices.
Wiegratz 2010	Multi-country: Germany, Austria	Quantitative, Cross-sectional	*n* = 1091 gynecologists	extended cycle OCPs	Gynecologists’ prescribing practices and personal experiences regarding extended cycle OCPs.
Woodsong 2014	Multi-country: Lilongwe, Malawi; Harare, Zimbabwe	Qualitative, In-depth interviews	*n* = 37 midwives, traditional healers, physicians, nurses, counsellors	vaginal gel for HIV and pregnancy prevention	Health Worker thoughts on vaginal gel for contraception and HIV prevention.
Yam 2007	Multi-country: Barbados; Kingston, Jamaica	Quantitative, Cross-sectional	*n* = 428 pharmacists, GPs, Ob/Gyn physicians, nurses	EC	Health Worker knowledge and thoughts on emergency contraception pills and their prescribing practices.

EC, emergency contraception; GP, general practitioner; IM, intramuscular; IUD, Intrauterine device; LARCs, long-acting reversible contraception; LNG-IUS, levonorgestrel-releasing intrauterine system; NP, nurse practitioner; OCPs, oral contraceptive pills; PAs, physician assistants; Sub-Q, sub-cutaneous.

Values and preferences of health workers on contraception methods varied by factors related to the contraceptive method itself, the contraceptive user, and the health worker. Below, we present findings from the included studies by these 3 main factor categories.

### 3.2 Contraceptive methods

Multiple articles found that specific contraceptive method characteristics affected values and preferences [[22], [23], [24],^26^,^28^,^29^,[31], [32], [33], [34], [35], [36],^40^,^49^,^52^,[54], [55], [56]]. Contraceptive method characteristics discussed in these articles included efficacy, safety, effect on the menstrual cycle and bleeding pattern, contraindications, convenience, good and adverse side effects, visibility of the method, ease of use, frequency of intake or administration, method of administration (e.g. barrier, oral, patch, ring, insertion, injection), duration of use, onset of action, ease of removal or discontinuation, STI prevention, accessibility and affordability, and time to return to fertility.

Four studies from Australia [29,33], Sweden [34], and the United Kingdom [53] found that a contraceptive method's effect on menstrual periods and bleeding could be taken as a positive or negative side effect, depending on the contraceptive user's preferences, and often influenced health worker preferences and counseling. Two cross-sectional studies from high-income countries mentioned preferences of methods for lactating women [20,24]: 1 study from the United States found that postpartum nurses did not prefer provision of medroxyprogesterone intramuscular injection in postpartum women due to their personal beliefs regarding its effect on lactation and decrease in milk production, despite receiving training on its benefits [20], and another study from Germany found that health workers more often recommended lactating women use progesterone-only contraceptive methods like POP and LNG-IUD [24].

Some articles described specific preferences or dislikes by health workers for certain contraceptive methods. Natural family planning methods discussed in 3 studies from Canada [27], Russia [36] and Australia [33] found that these methods were often not mentioned to clients as some health workers had reservations regarding their efficacy. However, other health workers better understood the efficacy of natural family planning or even preferred natural family planning methods, especially due to their non-hormonal nature and preferred use by clients who could not take hormones [33,51]. Additionally, a qualitative study from Nigeria found that the religious and cultural acceptability of natural family planning in Nigeria played a role in health workers recommending this to contraceptive users who did not want to use other forms of contraception [51]. Condoms were recommended because of their perceived safety profile by health workers in the United Kingdom [53] and Kenya [42]. One qualitative study from the United States discussed female condoms in particular: this study found variable knowledge on the method, and that health workers liked the spontaneity and control associated with the method but considered the design of the condom a barrier to its use [39]. Two cross-sectional studies in the United States and Germany/Austria discussed the acceptance and counseling on use of extended COCPs: most health workers approved its use but expressed varied methods of counseling, including how many weeks of active pills to recommend, when to start, and how to manage bleeding patterns with extended use [47,55]. IUDs were noted to be risky in Russia by health workers if the contraceptive user had multiple partners due to increased risk of STIs [36]. However, one cross-sectional study noted that other health workers counseled on IUDs and considered the method safe [38]. Male methods such as vasectomy were explicitly mentioned in 2 studies from France and the United States: in general, male methods were not counseled on as often as contraceptive methods for females, and when compared to female sterilization, male sterilization was counseled on less frequently, though 57% of Fellowship in Family Planning (Ob/Gyn and family medicine physician trainee) respondents in the US reported that vasectomy was their preferred method of sterilization [19,43].

Two qualitative studies contrasted preferences between newer and older contraceptive methods: the study from Russia found that health workers preferred the newer modern methods over the older methods [36], whereas the study from Australia mentioned the cost for newer pills was prohibitive to their use compared to older generation pills, and thus was a negative attribute of the newer pills [33].

### 3.3 Contraceptive user

Values and preferences for a method and its provision by health workers were often influenced by factors specific to the contraceptive user. Demographic information such as age, marital status, parity, literacy level, and medical diagnoses were mentioned in numerous articles as influential factors [21,31,33,34,36,38,39,45,46,50,53,57]. Three cross-sectional studies [21,38,46] and 1 qualitative study [33] from the United States [38,46], Switzerland [21], and Australia [33] found that a contraceptive user's medical history influenced a health worker's thoughts on an ideal method, contraindications to a method, or more specifically the dose of COCPs prescribed. Health workers also recommended specific methods based on an individual's desired timing of future pregnancies or decision to complete childbearing altogether [30]. One cross-sectional study from Germany found that a health worker's preferred contraceptive method for a specific client may be influenced by their perception of the client's preference and that this did not always align with the method that the client desired [44].

Numerous studies noted that health workers had varied preferences for methods with regards to young people/teenagers [31,36,38,39,45,53] or unmarried women [36,38,45] when compared to “mature” or married women. Six studies from the United States [31,38,50], Sweden [34], the United Kingdom [53], and Uganda [45] found that preferences for methods were affected by whether a contraceptive user was nulliparous, a teenager, or of younger age. Eight studies mentioned that certain methods were preferred based on age and the health worker's perceptions of the individual client's risk and needs at that age [31,33,38,39,45,46,53,57]. Four studies from the United Kingdom [53], Uganda [45], Russia [36], and the United States [39] found that health workers preferred abstinence, male and female condoms (for double-protection against both pregnancy and sexually transmitted infections (STIs)), and shorter-acting methods for young people.

With regard to long-acting reversible contraceptives (LARCs) for young people, 1 cross-sectional study from the United States found that health workers felt young people may be at higher risk for STIs or non-monogamous relationships, or that long-acting contraceptive methods could encourage young people to engage in more sexual activity [38]. Specifically for IUDs, some health workers thought IUDs were less appropriate or more difficult to place for nulliparous women [31,34,38,50], whereas others felt comfortable placing an IUD in a nulliparous woman due to its high efficacy in preventing pregnancy [38,53]. Additionally, a study from Uganda found that health workers were worried about the effect of taking hormones at a younger age on fertility [45].

For emergency contraception, 2 cross-sectional studies found that a health worker's preferences for emergency contraception and provision to specific clients was sometimes influenced by the contraceptive user's circumstances, such as being a victim of rape, being in a committed relationship, their age and/or need for parental consent, use of emergency contraception repeatedly in the past, and whether the contraceptive user or partner was requesting the medication [41,57]. One mixed-methods study from Kenya discussing contraception in individuals living with HIV found that health workers recommended dual protection (condoms plus another method) for prevention of both HIV/STIs and pregnancy with male clients, but not specifically with female HIV clients. Additionally, they preferred female sterilization for HIV-positive men or women who were done with childbearing [44].

### 3.4 Health workers

Factors related to the health workers themselves also influenced their values and preferences for contraceptive methods. In general, studies demonstrated that a health worker's comfort level, contraceptive knowledge, experience, depth of training, and time since training or graduation [17,18,29,34,38,39,42,43,45,48,57] affected values and preferences. Misconceptions about indications and safety of a method were influential: several studies discussed misconceptions about LARC guidelines or concerns for method safety in certain groups [29,31,38,45,50]. One study from Australia [29] and 2 studies from the United States [31,50] found that health workers had outdated information and perceived young people and nulliparous individuals ineligible for IUDs due to reproductive anatomy and safety concerns. A qualitative study from Uganda found that health workers had misconceptions that IUDs could result in infertility or cancer and were cautious with most clients, but especially with young or nulliparous clients due to infertility concerns [45]. A cross-sectional study from the United States demonstrated that health workers had concerns that the IUD increased risk of pelvic inflammatory disease (PID) and that someone with the history of STI or PID was not eligible for the device; this study found that the IUD was not offered by these health workers to this particular group of clients based on outdated information [38].

Demographics of health workers were also associated with differences in counseling. For example, a study from Canada found that natural family planning methods were more often discussed by religious, older age, female health workers, and those who practiced family medicine [27]. Five studies from multiple countries around the world demonstrated that age, years out of residency for physicians, and time in practice and type of practice affected the depth of counseling, knowledge and/or misconceptions regarding certain methods, and provision of methods such as LARCs [29,30,46,50,54]. These studies discussed counseling/provision of methods and were included in the review as they were interpreted to be reflective of health worker preferences. A qualitative study from Sweden found that recommendation for contraceptive methods could be influenced by the health worker's environment, colleagues’ experiences, and local culture [34]. Two qualitative studies described how some health workers had clear preferences for certain methods and used their knowledge and authority to influence a contraceptive user's decision [33,36].

Three cross-sectional studies from the United States [41], South Korea [37], and Barbados/Jamaica [57] discussed emergency contraception in particular. Two of these studies mentioned that health workers' preferences for EC were affected by religious and personal beliefs, clinical experience, and their education. Additionally, some health workers were not up-to-date on general knowledge, safety, and contraindications of the method itself, which also affected preferences for provision of the method [41,57]. Two of the 3 studies discussed health workers' thoughts on prescription vs over-the-counter availability of emergency contraception, with varied levels of support and rationale noted between different types of health workers [37,57]. The study from South Korea found that most health workers preferred prescribed provision of emergency contraception as they were concerned about the possibility for abuse of the method, believed that clients should receive counseling prior to use, or had safety concerns; however, those who preferred over-the-counter provision cited clients' rights and autonomy, increased access, minimal side effects, and decreased medical expenses as rationale [37].

### 3.5 Differences between types of health workers

Several studies examined differences around values and preferences for contraception between types of health workers [27,30,47,50,57]. Although some of these studies discuss counseling and provision, we have included them as reflective of the health worker preferences. Two studies, 1 in the United States [50] and the other a ten-country survey [30], reported differences between Ob/Gyn and Family Medicine/General Practitioners, where Ob/Gyns were more likely to offer IUDs and deem them safe when compared to Family Medicine/General Practitioners [30,50]. With regards to natural family planning, a cross-sectional study from Canada found that Family Medicine/General Practitioners were more likely to mention these methods during counseling when compared to Ob/Gyns [27]. Two cross-sectional studies compared Ob/Gyns to other health workers in general; the study from the United States found that Ob/Gyns were more likely to offer extended COCPs [47] while the study from Barbados/Jamaica noted that Ob/Gyns were most liberal with EC provision followed by GPs, nurses, and lastly pharmacists who were noted to be most conservative [57].

## 4 Discussion

This systematic review sought to examine health workers’ values and preferences for contraceptive methods. We identified 41 articles that discussed health worker values and preferences and found 3 specific influences: (1) contraceptive method characteristics, (2) the demographics, preferences, and desires of the contraceptive user, and health worker's perceptions of the client's preferences, and (3) factors related to the health worker themselves, such as training, knowledge, and comfort around contraception.

We noted a wide variety in values and preferences on contraceptive methods across the various specialties and types of health workers globally. Specific characteristics of contraceptive methods were found to be influential on a health worker's values and preferences. For example, the innate characteristics of methods, along with positive and negative side effects, ease of use, and cost, all influenced health worker preferences for methods. Preferences varied globally; this was highlighted more often for methods such as natural family planning, IUDs, emergency contraception, and male methods (i.e., vasectomy), often due to cultural acceptability of the method and health worker knowledge and/or misconceptions regarding method characteristics.

Health worker preferences for methods affect counseling and provision to clients; thus, it is important for health workers to be aware of clients’ preferences regarding specific contraceptive characteristics, so that contraceptive provision is patient-centered. Many clients value input from their health worker and thus shared decision-making in family planning is increasingly recognized as a valuable approach. It has been associated with improved client satisfaction with counseling compared with health worker-driven decision-making, and improved client satisfaction with their contraceptive method compared with client-driven decision-making [58].

Additionally, in line with findings from other reviews [3], we found studies from multiple countries documenting health worker biases towards certain contraceptive user populations, particularly young people (e.g., teenagers), unmarried individuals, or those who have never been pregnant. Often such biases are based on inaccurate factual information or social norms that stigmatize sexual activity among youth or other specific groups [3]. They thus violate established World Health Organization (WHO) guidance on contraception and human rights [59], which requires non-discriminatory service provision. Moreover, these views limit the provision of comprehensive, rights-based care to all women of childbearing age and appear to be related to health worker education and training, experience, environment, and comfort with contraceptive provision.

Notable differences in counseling/provision and comfort around contraception were also seen between specialties and types of health workers. The time since a health worker's initial school and/or training affects their values, preferences, and counseling. Our review illustrates that health workers with continued experience or those more recently graduated generally feel more comfortable with contraceptive provision, including LARCs. This highlights the importance of continued education around contraception and counselling/communication skills with an emphasis on updated guidelines, client-centered care, and refresher courses on procedures such as LARC insertion and removals. Health organizations and schools can offer continued education courses and workshops to keep health workers up-to-date on contraceptive changes and also create a network of health workers who they can rely on for support and questions once out of their training period.

Strengths of this systematic review are the inclusion of studies from numerous countries and diverse settings. We included qualitative, quantitative and mixed/multi-methods studies and covered a wide range of contraceptive methods, including hormonal methods, male methods, and natural family planning. Articles in this review included perspectives from a variety of health workers, from physicians to nurses, pharmacists, and community health workers. Limitations of the review include that this was part of a larger review; thus we may have missed potentially relevant articles. Specifically, we note that this review was not designed to comprehensively review the evidence base for contraceptive counseling overall – including how end-users are counseled about contraceptive methods or health workers' preferences in how to provide contraceptive counseling. Other recent reviews summarize the literature on the impact of contraceptive counseling provided in clinical settings [60], client preferences for contraceptive counseling [61], and the effectiveness of counseling strategies for modern contraceptive methods [62]. Additional research has been conducted on how physicians' characteristics are correlated with their recommendations for specific contraceptive methods for standardized patients [63]. Furthermore, the review found poor representation from low-resource settings: we identified only 10 studies conducted in low or lower-middle income countries. Due to the variety of health workers who have received different types of education and training, it may be difficult to generalize findings across types of health workers. Lastly, although all contraceptive methods were represented in this review, we found limited studies on emergency contraception and natural family planning and a higher number of studies on LARCs and COCPs. This may be due to higher provision of certain contraceptive methods over others, and greater attention to LARCs in recent years.

This systematic review highlights that health worker values and preferences for contraceptive methods are influenced by multiple factors including their background and environment. Future work should address gaps in health worker knowledge and training and work to reduce overly restrictive perspectives that health workers hold regarding certain contraceptive methods and populations. The health care community can continue providing a support and training network to itshealth workers to ensure high-quality, rights-based, and patient-centered contraceptive services. Future research should focus on how to improve and standardize education/training for all types of health workers within their scope of practice and to better understand and address harmful biases that health workers may have towards certain populations.

## Acknowledgments

This review was supported by the World Health Organization, Department of Sexual and Reproductive Health and Research. We would like to thank the 2014 WHO MEC Guideline Development Group for their inputs and the Johns Hopkins Bloomberg School of Public Health graduate students who contributed to the screening and data abstraction process.

## Appendix Supplementary materials

Image, application 1

Image, application 2
