# Supplementary material for: Health workers’ values and preferences regarding contraceptive methods globally: A systematic review
Source: Contraception. 2022 Jul;111:61–70. doi: 10.1016/j.contraception.2022.04.012 (PMC9233149; doi:10.1016/j.contraception.2022.04.012)
Supplement: Supplementary file 1 [file mmc1.docx]

**Appendix 1. Risk of bias assessment for qualitative studies or studies with a qualitative component presenting values and preferences of health workers**

| **Lead author year** | **Clear statement of research aims** | **Qualitative methodology appropriate** | **Research design appropriate** | **Recruitment strategy appropriate** | **Data collected in a way that addressed the research issue** | **Adequately considered researcher-participant relationship** | **Ethical issues considered** | **Data analysis sufficiently rigorous** | **Clear statement of findings** |
| --- | --- | --- | --- | --- | --- | --- | --- | --- | --- |
| Akers 2010 | Yes | Yes | No | Yes | Yes | No | Yes | Yes | Yes |
| Alem 2014 | Yes | Yes | No | Yes | Yes | Yes | Yes | Yes | Yes |
| Brunie 2019 | Yes | Yes | Yes | Yes | Yes | Yes | Yes | Yes | Yes |
| Burke 2014 | Yes | Yes | Yes | Yes | Yes | Yes | Yes | Yes | Yes |
| Callahan 2019 | Yes | Yes | Yes | Yes | Yes | Yes | Yes | Yes | Yes |
| Garrett 2015 | Yes | Yes | Yes | Yes | Yes | No | Yes | Yes | Yes |
| Kavanaugh 2013 | Yes | Yes | Yes | Yes | Yes | No | Yes | Yes | Yes |
| Keith 2014 | Yes | Yes | Yes | No | Yes | No | Yes | Yes | Yes |
| Kelly 2016 | Yes | Yes | Yes | Yes | Yes | No | No | Yes | Yes |
| Kilander 2017 | Yes | Yes | Yes | Yes | Yes | No | Yes | Yes | Yes |
| Larivaara 2010 | Yes | Yes | Yes | Yes | Yes | Yes | Yes | Yes | Yes |
| Mantell 2011 | Yes | Yes | Yes | Yes | Yes | No | Yes | Yes | Yes |
| McLean 2017 | Yes | Yes | Yes | Yes | Yes | Yes | Yes | Yes | Yes |
| Newmann 2013 | Yes | Yes | Yes | Yes | Yes | Unclear | Yes | Yes | Yes |
| Paul 2016 | Yes | Yes | Yes | Yes | Yes | No | Yes | Yes | Yes |
| Sweeney 2015 | Yes | Yes | Yes | Yes | No | No | Yes | Yes | Yes |
| Tolley 2014 | Yes | Yes | Yes | Yes | Yes | No | No | No | Yes |
| Ujuju 2011 | Yes | Yes | No | Yes | Yes | No | Yes | No | No |
| Wiebe 2012 | Yes | Yes | Yes | Yes | Yes | No | Yes | yes | Yes |
| Woodsong 2014 | Yes | Yes | Yes | Yes | Yes | Yes | Yes | Yes | Yes |
