# Supplementary material for: Health workers’ values and preferences regarding contraceptive methods globally: A systematic review
Source: Contraception. 2022 Jul;111:61–70. doi: 10.1016/j.contraception.2022.04.012 (PMC9233149; doi:10.1016/j.contraception.2022.04.012)
Supplement: Supplementary file 2 [file mmc2.docx]

**Appendix 2. Risk of bias assessment for quantitative studies or studies with a quantitative component presenting values and preferences of health workers**

| **Lead author, year** | **Cohort** | **Control or comparison group** | **Pre/post intervention data** | **Random assignment of participants to intervention** | **Random selection of participants for assessment** | **Follow-up rate >= 80%** | **Comparison groups equivalent at baseline in socio-demographics** | **Comparison groups equivalent at baseline in outcome measures** |
| --- | --- | --- | --- | --- | --- | --- | --- | --- |
| Amouroux 2018 | No | No | No | NA | No | NA | NA | NA |
| Benfield 2018 | No | No | Yes | NA | No | NA | NA | NA |
| Bitzer 2009 | No | No | No | NA | Yes | NA | NA | NA |
| Bombas 2012 | No | No | No | NA | No | NA | NA | NA |
| Buhling 2014 | No | No | NR | NR | No | No | NR | NR |
| Callahan 2019 | No | No | No | NA | Yes | NA | NA | NA |
| Choi 2010 | No | No | No | NA | Yes | NA | NA | NA |
| Donnelly 2014 | No | No | No | No | No | NA | NA | NA |
| Gemzell-Danielsson 2012 | No | No | No | NA | Yes | NA | NA | NA |
| Knox 2012 | No | No | No | NA | No | NA | NA | NA |
| Lee 2019 | No | No | No | NA | No | NA | NA | NA |
| Madden 2010 | No | No | No | No | No | NA | NA | NA |
| McLean 2017 | No | No | No | No | No | No | No | No |
| Munsell 2009 | No | No | No | No | No | NR | NR | NR |
| Newmann 2013 | No | No | No | NA | No | NA | NA | NA |
| Nguyen 2017 | No | No | No | No | No | No | No | No |
| Oppelt 2017 | No | No | No | No | No | No | No | No |
| Philliber 2014 | No | Yes | No | No | No | No | No | No |
| Sulak 2006 | No | No | No | NA | No | NA | NA | NA |
| Tyler 2012 | No | No | No | NA | Yes | NA | NA | NA |
| Weisberg 2013 | No | No | No | NA | No | NA | NA | NA |
| Wellings 2007 | No | no | No | NA | NA | NA | NA | NA |
| Wiebe 2012 | No | No | No | NA | No | NA | NA | NA |
| Wiegratz 2010 | No | No | No | NA | NR | NA | NA | NA |
| Yam 2007 | No | No | No | No | No | NA | NA | NA |

NA: not applicable; NR: not reported
